# Retinal Detachment in the Setting of Neurofibromatosis Type 1

**DOI:** 10.1177/24741264251374596

**Published:** 2025-10-17

**Authors:** Lauren Pickel, Miguel Cruz Pimentel, Anarsaikhan Narmandakh, Austin Pereira, Peng Yan

**Affiliations:** 1 Temerty Faculty of Medicine, University of Toronto, Toronto, ON, Canada; 2 Department of Ophthalmology and Vision Sciences, University of Toronto, Toronto, ON, Canada; 3 Kensington Eye Institute, Toronto, ON, Canada

**Keywords:** neurofibromatosis type 1, NF1, retinal detachment, retinal dialysis, von Recklinghausen

## Abstract

**Purpose:** Neurofibromatosis type 1 (NF1) is a multisystem neurocutaneous syndrome that includes ocular manifestations. This systematic literature review aimed to examine evidence of an association between NF1 and retinal detachment (RD). **Methods:** Ovid MEDLINE, EMBASE, and PubMed were searched from database inception to February 2024 for reports of RD related to NF1. An additional case of spontaneous RD in a young patient with NF1 is reported. **Results:** In total, 27 reported cases of NF1-associated RD were identified, of which 14 were associated with intraocular or intraorbital space-occupying lesions. Lesion-associated RDs were exudative and presented in patients at a median age of 19.6 years (range 10 to 36 years). The remaining 13 cases, and the novel case reported herein of a patient with RD secondary to a giant retinal tear, were spontaneous rhegmatogenous RD, presenting in patients at a median age of 19.1 years (range 22 months to 49 years). The most common presentation of spontaneous RD was an asymptomatic finding on routine exam (55% of reported cases). **Conclusions:** NF1 may increase the risk of RD through 2 mechanisms: exudation caused by space-occupying intraorbital lesions, or rhegmatogenous RD resulting from irregularities in vitreoretinal adhesion due to abnormal collagen production. While further evidence is needed, extended ocular screening of patients with NF1 into adulthood may be considered.

## Introduction

Neurofibromatosis type 1 (NF1) is an autosomal-dominant neurocutaneous syndrome caused by mutation in the *NF1* tumor suppressor gene.^1^ While penetrance is complete, expression is variable, with multisystem manifestations contributing to the clinical diagnosis of NF1, including optic pathway glioma and iris Lisch nodules.^1^ Other, nondiagnostic ocular manifestations of NF1 include neurofibromas of the conjunctiva, glaucoma, ectropion uveae, myopia, periorbital plexiform neurofibromas, astrocytic hamartomas, capillary hemangiomas, and choroidal^2^ and retinal vascular abnormalities.^3^ Additionally, though not part of the original diagnostic criteria, choroidal nodules have been detected in the majority of children and adults with NF1 and have been proposed as a novel diagnostic criterion.^4^ A recent revision of the National Institutes of Health diagnostic criteria for NF1 and Legius syndrome includes choroidal nodules as an alternative to iris Lisch nodules.^1^

Current ocular screening recommendations for NF1 propose that children should be screened for the presence of optic pathway gliomas.^5^ It is recommended that children with NF1 have annual eye exams, including dilated fundus exams, until the age of 8 years, then biannually until adulthood.^6^ While there is variation in screening practices among different centers, screening typically ceases at ages 16–18 years.^7^ However, recent case descriptions suggest a link between NF1 and retinal detachment (RD), occurring through adolescence and into adulthood. We present a comprehensive systematic literature review of cases of RD in individuals with NF1 and offer an additional case of RD secondary to a giant retinal tear. The pathophysiology of RD in the setting of NF1, as well as implications for screening, are further discussed.

## Methods

A systematic literature review was performed to search Ovid MEDLINE, EMBASE, and PubMed from database inception to February 2024. All citing articles and reference lists of relevant existing reports were manually scanned for additional reports of NF1-associated RD. Exclusion criteria were articles without full-text availability, articles not available in English, and publications that were not peer-reviewed. There was no restriction based on study design; case studies and case series were included. The following search terms were used: *neurofibromatosis 1* or *von Recklinghausen disease* or *NF1* and *retinal detachment* or *detached retina* or *retinal break* or *retinal tear*. Cases were categorized as RD associated with intraorbital space-occupying lesions, which may plausibly have contributed causally to their formation, or otherwise as spontaneous RD. A novel case of giant retinal tear–associated RD and retinal dialysis in the contralateral eye is additionally presented.

## Results

The literature search identified 16 distinct reports of NF1-associated RD in 27 patients. Of these cases, 14 were associated with an intraorbital space-occupying lesion (Table 1). One case was initially thought to be associated with ocular trauma, but retinal capillary hemangiomas were later found on exam.^8^ The remaining 13 cases identified in the literature search (Table 2) and the additional case of RD reported below were thought to be spontaneous RD without associated space-occupying lesions or trauma. The median age at time of presentation was 19.6 years (range 10 to 36 years) for those with lesion-associated RD, 19.1 years (range 22 months to 49 years) for those with spontaneous RD, and 19.3 years overall. Among the studies where sex was reported, there were 13 male patients and 10 female patients.

**Table 1.** Cases of Retinal Detachment in NF1 Associated With Space-Occupying Lesions.

Reference	Case(s); Age/Sex; Diagnosis	Presentation	Mass	Retina Findings	Other Ocular Findings	Non-Ocular Findings of NF1
Shields et al, 2013^9^	n = 5; age NR; known NF1	NR	Retinal vasoproliferative tumor	RD	NR	NR
Oystrek et al, 2012^10^	n = 1; 14-year-old male; known NF1	Referred for fundus mass; VA 20/20 OD and 20/50 OS	Retinal vasoproliferative tumor	Exudative RD (inferotemporal)	None	Café-au-lait spots
Oystrek et al, 2012^10^	n = 1; 36-year-old male; known NF1	Referred for fundus mass; VA LP OD and 20/20 OS	Retinal vasoproliferative tumor	Total RD of enucleated eye	Iris Lisch nodules; conjunctival hyperemia; corneal edema; NVI; ectropion iridis	Café-au-lait spots; neurofibromas; axillary freckling
Oystrek et al, 2012^10^	n = 1; 28-year-old female; known NF1	Painless vision loss and mass; VA HM OD and 20/20 OS	Optic nerve glioma	Exudative RD (inferior)	None	Café-au-lait spots; neurofibromas
Asadi-Amoli and Tabatabaei, 2007^11^	n = 1; 20-year-old male; known NF1	Congenital blindness and orbital deformity; VA NR	Choroidal hamartoma and plexiform neurofibroma	Exudative RD	NR	NR
Destro et al, 1991^8^	n = 1; 10-year-old female; undiagnosed	Symptoms NR; VA 20/70 OD and 20/40 OS	Retinal capillary hemangiomas	RD (superonasal) with dialysis	None	Café-au-lait spots; neurofibromas
Destro et al, 1991^8^	n = 1; 15-year-old male; known NF1	Sudden monocular vision loss, pain and photophobia; VA 20/100 OD and 20/20 OS	Peripheral astrocytic hamartoma	RD (superior) with dialysis	NVI; iritis; IOP 40 mm Hg	Café-au-lait spots
Destro et al, 1991^8^	n = 1; 16-year-old male; undiagnosed	3 months monocular vision loss; VA LP OD and 20/20 OS	Large astrocytic hamartoma	RD with dialysis	None	Café-au-lait spots; neurofibroma
Destro et al, 1991^8^	n = 1; 25-year-old female; known NF1	Acute monocular vision loss; VA 20/20 OD and HM OS	Combined hamartoma of the retina and RPE	Total RD with large tear (inferotemporal far periphery)	Iris Lisch nodules	Café-au-lait spots; neurofibromas; axillary freckling; skeletal deformity (leg)
Martyn and Knox, 1972^12^	n = 1;12-year-old female; known NF1	3 weeks redness, discomfort, and recent vision loss; VA LP OD and 20/15 OS	Glial hamartoma	RD (supratemporal) with subretinal hemorrhage	Glaucoma, refractory	Café-au-lait spots; neurofibromas

Abbreviations: HM, hand motions; LP, light perception; NF1, neurofibromatosis type 1; NR, not reported; NVI, neovascularization of the iris; RD, retinal detachment; RPE, retinal pigment epithelium; VA, visual acuity.

**Table 2.** Cases of Spontaneous Retinal Detachment in NF1.

Reference	Case; Age/Sex; Diagnosis	Presentation	Retina Findings	Other Ocular Findings	Non-Ocular Findings of NF1	Treatment; VA Outcome
Boulanger et al, 2024^13^	6-year-old male; known NF-1	NR; VA NR	Total RD with posterior tear	NR	NR	PPV, scleral buckling, and silicone oil tamponade; VA NR
Shah et al, 2024^14^	21-year-old female; known NF1	Decreased vision for 6 months; VA 20/200 OD and 20/25 OS	RRD 9:00–5:00 OD; dialysis with pigmentary demarcation 10:00–2:30 OD	Iris Lisch nodules; choroidal nodules	Café-au-lait spots; congenital pseudoarthritis of the tibia	Scleral buckling, cryopexy, and PnR; VA 20/100 OD at 1 year
Shah et al, 2024^14^	20-year-old female; known NF1	Blurry vision for 8 months; VA 20/20 OD and 20/400 OS	Near total RRD, sparing inferior periphery; dialysis 10:00–11:00 OS	Iris Lisch nodules; choroidal nodules; history of optic glioma (contralateral)	Café-au-lait spots; congenital pseudoarthritis of the tibia	Scleral buckling, cryopexy, and PnR; VA 20/150 OS at 5 years
Shah et al, 2024^14^	21-year-old male; known NF1	Asymptomatic on routine exam; VA 20/30 OD and 20/20 OS	RRD 2:00–7:00 OD; dialysis 4:00–6:00 OS, retina attached	Iris Lisch nodules; choroidal nodules	Café-au-lait spots; pilocytic astrocytoma	Scleral buckling, cryopexy, external drainage, and PnR OD, and laser retinopexy OS; VA 20/20 OU at 3 months
Shah et al, 2024^14^	14-year-old male; known NF1	Failed school vision screening; VA 20/25 OD and 20/400 OS	Macula-off RRD 2:00–10:00 OS; dialysis 3:00–5:00 OS	History of optic nerve gliomas OU	Café-au-lait spots; cutaneous neurofibromas	Scleral buckling, cryopexy, external drainage, and PnR; VA 20/200 OS at 5 years
Geethika et al, 2023^15^	18-year-old female; known NF1	Asymptomatic on routine exam; VA 20/60 OD and 20/40 OS	Macula-on inferior RD; 2–3 retinal holes	Iris Lisch nodules; high myopia; plexiform neurofibromatosis of lid (contralateral)	NR	Scleral buckling and cryopexy; VA NR
Hua et al, 2023^16^	NR	Clinical trial, reported as AE unrelated to study intervention; VA NR	RRD	NR	NR	NR
Shrestha et al, 2022^17^	36-year-old male; known NF1	Asymptomatic on screening; VA 20/30 OD and 20/400 OS	Macula-off RRD (inferior); dialysis 4:00–7:00	Exotropia (8-degree prism diopter); positive RAPD	Café-au-lait spots; cutaneous neurofibromas; pleural effusion	NR
Hua et al, 2021^18^	22-month-old female; undiagnosed	Leukocoria, pain, inflammation, exotropia for 1 month	Total funnel RD with cystic formation	Small-globe conjunctival injection; IOP 37 mm Hg; lack of patent vasculature on FA	Large supraglottic plexiform neurofibroma; gliomatosis cerebri	Deemed inoperable, sub-Tenon Kenalog injection, topical prednisolone, and glaucoma medications; VA NR
Clemente-Tomas et al, 2020^19^	13-year-old male; known NF1	Asymptomatic on annual exam; VA 20/20 OU	Macula-on RRD; superonasal dialysis	Iris Lisch nodules	NR	PPV, scleral buckling, cryopexy, and endophotocoagulation; VA NR
Kilgore et al, 2020^20^	39-year-old female; known NF1	Visual field loss (temporal defect) for 2 months following an absence seizure; VA: 20/20 OU	Macula-on RRD inferonasal; dialysis superonasal and inferotemporal	Iris Lisch nodules	Café-au-lait spots; cutaneous neurofibromas	Lost to follow-up
Oktenli et al, 2003^21^	20-year-old male; known NF1	Referred for dysmorphic features; VA 20/20 OD and 20/25 OS	RD with pigmentary demarcation; superonasal hole at 8:00 OS	Iris Lisch nodules	Café-au-lait spots; cutaneous neurofibromas; multiple dysmorphic features of face and limb	NR
Van Es et al, 1996^22^	8-year-old; known NF1	Sudden vision loss; VA NR	RD	NR	NR	NR

Abbreviations: AE, adverse event; FA, fluorescein angiography; HM, hand motions; IOP, intraocular pressure; LP, light perception; NF1, neurofibromatosis type 1; NR, not reported; PnR, pneumatic retinopexy; PPV, pars plana vitrectomy; RAPD, relative afferent pupillary defect; RD, retinal detachment; RRD, rhegmatogenous retinal detachment; VA, visual acuity.

Visual acuity at presentation varied from 20/20 to light perception. The most common presentation of spontaneous RD in NF1 was as an asymptomatic finding on routine exam (6 of 11 reported cases), followed by painless gradual vision loss (3 of 11 reported cases). Spontaneous RD presented with sudden vision loss in 1 case, and in a case describing a 22-month-old patient, spontaneous RD presented as pain and leukocoria. In cases of lesion-associated RD, vision loss was the most common presenting feature, being variably painless and gradual (2 of 8 cases), painless and sudden (1 of 8 cases), painful and sudden (2 of 8 cases), or congenital (1 of 8 cases). Two cases presented with asymptomatic masses seen on fundus exam. Remaining reports did not include descriptions of presenting symptoms. Notably, RD was the presenting feature of previously undiagnosed NF1 in 3 cases (spontaneous RD in a 22-month-old patient, and lesion-associated RD in a 10-year-old patient and 16-year-old patient).

In cases of spontaneous RD, retinal exam findings suggested the presence of rhegmatogenous RD in 11 of 12 reports, with 1 of the cases being a total funnel RD with unknown initial mechanism. Retinal dialysis was observed in 7 cases (54%), with 1 being in the contralateral eye. Retinal holes were visualized in an additional 2 cases. There was no apparent pattern in quadrant of RD. Lesion-associated RDs were found overlying or adjacent to the respective lesion and more often appeared exudative.

In cases of RD associated with intraocular or intraorbital space-occupying lesions, indicated in reports that evaluated the suspected mechanism, most of the cases were thought to be exudative (5 of 9 cases). Exudative detachment was associated with retinal vasoproliferative tumors, optic nerve glioma, glial hamartoma, choroidal hamartoma, and plexiform neurofibroma. On the other hand, a single report^8^ described 4 cases of rhegmatogenous RD with retinal dialysis secondary to capillary hemangiomas or hamartomas.

Treatment and visual outcomes in cases involving space-occupying lesions varied greatly and were dependent on the nature of the lesion. Lesions included retinal vasoproliferative tumors presenting as painless vision loss and fundus mass; optic nerve gliomas presenting similarly; and hamartomas of the choroid, astrocytes/glia, or retina and retinal pigment epithelium presenting as vision loss with or without ocular pain. Vision loss varied in its onset from acute to insidious and was often severe. In cases of spontaneous RD, there was a partial return of visual acuity after treatment with combinations of scleral buckle, cryopexy, laser and pneumatic retinopexy, or pars plana vitrectomy with endophotocoagulation. Deficits in vision remained (visual acuity range 20/100 to 20/200) in all but 1 case, in which vision was largely spared pretreatment (visual acuity 20/30) and improved to 20/20.

## Case Presentation

**Figure 1.**
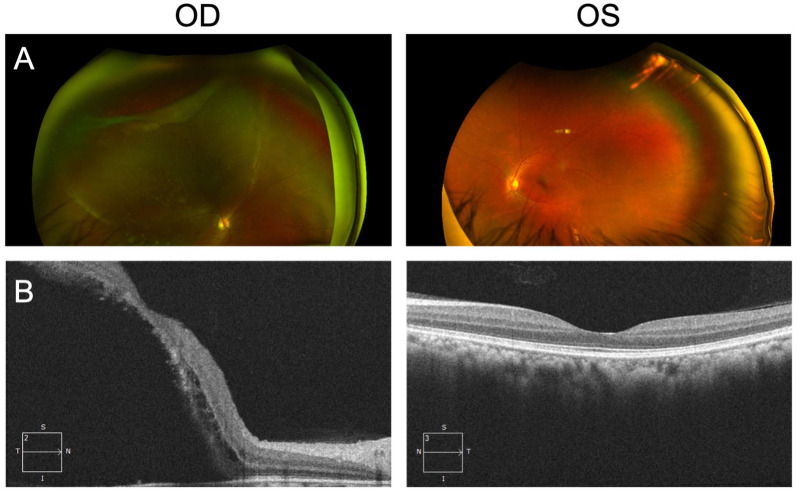
A 32-year-old woman with a history of NF1 presented with progressive vision loss. (A) Initial color fundus photographs of both eyes show rhegmatogenous retinal detachment involving the macula secondary to giant retinal tear OD (10:30 to 1:30 clock hours) and evidence of proliferative vitreoretinopathy with yellow-white deposits, indicating chronicity of the retinal detachment OD and retinal dialysis OS. (B) Initial optical coherence tomography images of both eyes show macula-off retinal detachment OD with a patchy (moth-eaten) appearance, indicative of photoreceptor outer segment loss.

A 32-year-old East Asian woman with a history of NF1 presented to our clinic with a 2–3-week history of progressive vision loss in the inferior visual field OD. There was no history of ocular trauma. Visual acuity on presentation was counting fingers OD and 20/20 OS. Intraocular pressure was 10 mm Hg bilaterally. A dilated fundus exam of her right eye showed a superior rhegmatogenous RD involving the macula, with a giant retinal tear extending from 10:30 to 1:30 clock hours, evidence of proliferative vitreoretinopathy grade B, and presence of yellow-white deposits on the retinal surface of the detached retina, indicating chronicity^23^ (Figure 1A). Optical coherence tomography confirmed the presence of macula-off, stage 5 RD with a patchy (moth-eaten) appearance,^24^ indicating loss of photoreceptor outer segments (Figure 1B). A dilated fundus exam of her left eye with scleral depression incidentally revealed retinal dialysis extending across 3:00 clock hours, without any reported symptoms.

**Figure 2.**
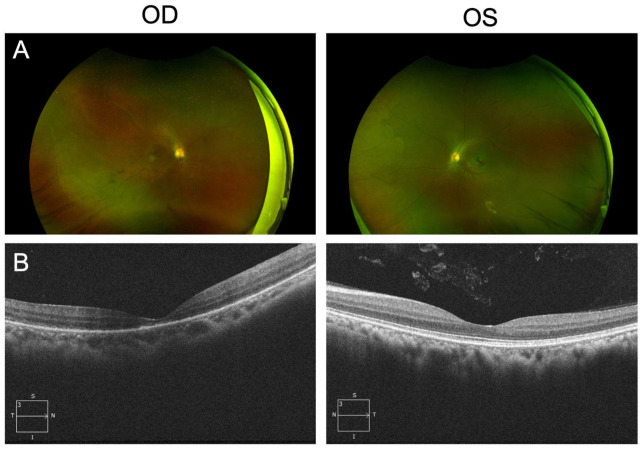
At 4 months’ follow-up in the 32-year-old woman diagnosed with retinal detachment secondary to a giant retinal tear, (A) color fundus photographs and (B) optical coherence tomography images of both eyes show the reattached right retina after the patient underwent pneumatic retinopexy OD and laser retinopexy OU.

The patient underwent an urgent pneumatic retinopexy with injection of 0.8 cc of pure SF6 gas and bilateral laser retinopexy. Upright head positioning was maintained for 1 week postprocedure. At the 4-month follow-up, the right retina remained reattached, with a well-formed laser retinopexy barricade around the giant retinal tear. However, macular and foveal atrophy was confirmed on optical coherence tomography. Additionally, the left retinal dialysis was well-contained within the laser retinopexy barricade (Figure 2, A and B). Visual acuity at the 4-month follow-up was 20/400 in the right eye and 20/20 in the left eye. Follow-up at 9 months showed stable visual acuity without recurrence of detachment.

## Discussion

An increasing number of reports link NF1 to risk of RD. This systematic review finds that while many cases of NF1-associated RD occur in the presence of intraocular space-occupying lesions, approximately half of the cases of RD in NF1 are spontaneous and rhegmatogenous in nature. Importantly, spontaneous RD most commonly presented as an asymptomatic finding (55% of reported cases) or as insidious painless vision loss on routine screening. Current screening guidelines in NF1 do not suggest routine ocular screening of adult patients. Moreover, although there is variation in actual screening practices, in a survey of centers with expertise in NF1, only 1 of 16 centers reported that screening was conducted without an age limit, with all remaining centers halting ocular screening in childhood or early adulthood, most commonly at age 16 or 18 years.^7^ In contrast, the mean age at the time of onset of spontaneous RD was 20 years, with multiple cases presenting in the second and third decade of life.

The risk of spontaneous RD in young individuals with NF1 is likely the result of abnormalities in the structure of the vitreous base, attributed to aberrant collagen production. Vitreoretinal adhesion depends on bundles of collagen fibrils which orient perpendicularly and insert through the basal lamina of the peripheral retina.^25^ In vitro studies of *Nf1*-mutant fibroblasts have demonstrated an aberrant proliferative response to epidermal growth factor and abnormal pattern of collagen arrangement, with increased production but disorganized deposition.^26–29^ Moreover, in the context of cutaneous neurofibromas, *Nf1* mutation results in reduced fibroblast expression of matrix metalloproteinase 1,^30^ an enzyme responsible for the remodelling and degradation of extracellular matrix components. The combination of overproduction of collagen proteins, disorganized deposition, and inadequate lysis would result in an irregular structure of collagen fibrils at the vitreous base.

Irregularities in vitreoretinal adhesion could increase susceptibility to RD through increased vitreoretinal traction in places of irregular adhesions, through reduced elasticity of the vitreous base which could make it more vulnerable to mechanical stress, or equally through areas of weakened vitreoretinal adhesion. In some cases, areas of weakened vitreoretinal adhesion may be secondary to vascular abnormalities, as in the case of a 6-year-old boy with a posterior retinal tear overlying neovascularization.^13^ There are also 2 reports in the literature of vitreous base avulsion occurring spontaneously in individuals with NF1.^31,32^ Vitreous base avulsion is seen almost exclusively in the context of blunt ocular trauma or surgery,^33^ but was observed bilaterally in a young woman with NF1 without history or evidence of ocular trauma or other cause.^31^ The bilateral presentation is particularly indicative of underlying structural abnormality. Likewise, the descriptions of a case of rhegmatogenous RD and contralateral retinal dialysis in a 14-year-old boy,^14^ the present case of RD associated with giant retinal tear and contralateral retinal dialysis in a 32-year-old woman, and the 13 other cases of spontaneous RD in young individuals with NF1 strongly suggest that compromised vitreoretinal adhesion is a manifestation of this common genetic condition.

An increased prevalence of space-occupying lesions may further contribute to an increased risk of RD in young patients with NF1. Intraocular lesions were observed in association with rhegmatogenous and exudative RDs. The mechanism of rhegmatogenous RD is likely to be a combination of abnormal vitreoretinal adhesion, as described above, with increased traction from the growing lesion. Exudative detachment occurs secondary to various intraocular neoplasms because of increased vascular permeability,^34^ and may in some cases have occurred secondary to intraorbital lesions if in sufficient proximity to the retina to induce exudation.

These suggested mechanisms are speculative, a key limitation of the present work. Limited information specific to each case is available, and therefore speculation regarding mechanism is not feasible. For instance, it is possible that intraorbital lesions and RD co-occurred spuriously without any causal relationship. In all cases, the potential contribution of the most common risk factors of RD—for example, high myopia in rhegmatogenous RD—must be considered, with NF1 not necessarily being the only or primary risk factor. Further work is needed to better understand the mechanisms of RD in NF1.

To our knowledge, this comprehensive review is the first systematic analysis of the clinical relationship between NF1 and RD in young individuals, with findings from all reports revealing a significant association. In approximately half of the reported cases, RD was attributed to space-occupying lesions within the eye, while the remainder resulted from spontaneous detachment. The *NF1* mutation can lead to abnormal collagen production, including overproduction, disorganized deposition, and reduced remodelling, which may result in irregular adhesions at the vitreoretinal interface, increasing the risk of retinal tears and detachment. The cases described herein demonstrate that, while ultra-widefield imaging can detect RD, it cannot detect asymptomatic dialysis. Therefore, clinicians should consider performing a scleral depression exam as an essential diagnostic test for young adults with NF1. It is imperative to conduct more extensive cohort studies to better understand the incidence of retinal tears and detachments in NF1. Given the mounting evidence supporting the association between NF1 and RD, it may be prudent to consider routinely screening adults with NF1 for RD.
